# Five Metagenome-Assembled Genomes of the Rare Phylum CSSED10-310 from Zodletone Spring (Oklahoma, USA)

**DOI:** 10.1128/MRA.00414-21

**Published:** 2021-07-01

**Authors:** Archana Yadav, C. Ryan Hahn, Mostafa S. Elshahed, Noha H. Youssef

**Affiliations:** a Department of Microbiology and Molecular Genetics, Oklahoma State University, Stillwater, Oklahoma, USA; Indiana University, Bloomington

## Abstract

We analyzed five metagenome-assembled genomes (MAGs) belonging to the rare, yet-uncultured phylum CSSED10-310 recovered from the anoxic sediments of Zodletone Spring (Oklahoma). Our analysis suggests their potential involvement in sulfite respiration.

## ANNOUNCEMENT

Zodletone Spring is a surficial, anoxic, sulfide- and sulfur-rich spring in southwestern Oklahoma. Prior studies have documented the phylogenetic diversity in the spring (1–5). Such studies have demonstrated that the spring harbors a plethora of novel and rare taxa. Here, we report on the assembly and analysis of five genomes belonging to the rare, yet-uncultured phylum CSSED10-310. Currently (April 2021), this phylum is represented in the Genome Taxonomy Database (GTDB; release 95) by a single genome (GCA_003558985.1) binned from sediments of a hypersaline soda lake (6). The phylum appears to be a sister phylum to the *Acidobacteriota*.

Samples from the anoxic, sulfide-saturated source sediments were obtained from Zodletone Spring in September 2017. Ten samples were collected from 5 cm deep into the anoxic sediments by completely filling sterile 50-ml polypropylene plastic tubes. The tubes were kept on ice until they were brought back to the lab (∼2-h drive), where they were immediately processed. DNA extraction was conducted on 0.5 g sediment from each of the 10 replicate samples using the DNeasy PowerSoil kit (Qiagen, Valencia, CA, USA) according to manufacturer’s protocols. All DNA extractions were pooled and used for the preparation of sequencing libraries using the Nextera XT DNA library prep kit (Illumina, San Diego, CA, USA) as per the manufacturer’s instructions. Sequencing was conducted using the Illumina HiSeq 2500 platform using the services of Novogene (Beijing, China), generating 281 Gbp of 150-bp paired-end raw sequence output. FastQC v0.11.5 (http://www.bioinformatics.babraham.ac.uk/projects/fastqc/) was used to assess the quality of the reads, followed by trimming using Trimmomatic v0.38 (7). High-quality reads were assembled into contigs using MEGAHIT v1.1.3 (8). MetaBAT 2 v1.7 (9) and MaxBin 2 v2.2.4 (10) were used to bin the contigs into draft genomes, and DasTool v1.1.1-0 (11) was used to select the highest-quality bins. Genome completeness, strain heterogeneity, and contamination were estimated using CheckM v1.1.3 (12). Default parameters were used except where otherwise noted. GhostKOALA (13) was used for functional annotation by assigning protein-coding genes to KEGG orthologies (KOs). KEGG mapper (14) was used to visualize metabolic pathways for this phylum. The taxonomic affiliation of the genomes was determined using GTDB-Tk v1.1.0 (15, 16), and the generated concatenated alignment was used to construct a maximum likelihood phylogenomic tree using FastTree (17).

**FIG 1:**
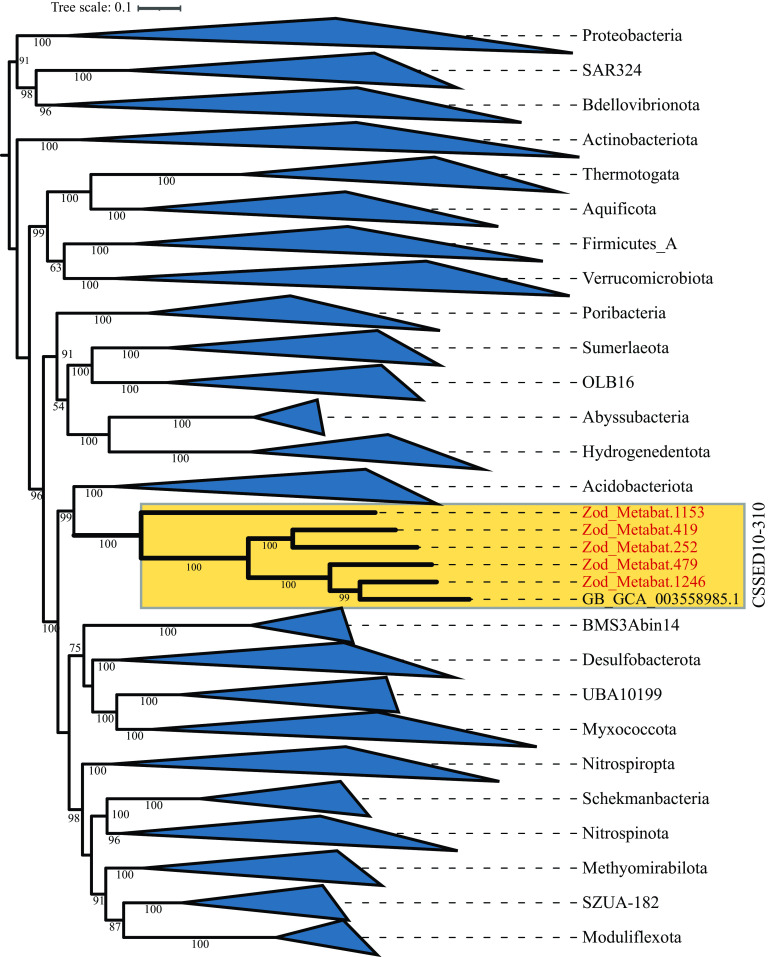
Maximum likelihood tree based on the concatenated alignment of 120 single-copy marker genes showing the phylogenetic position of phylum CSSED10-310 relative to other phyla. The tree was constructed in FastTree (17) and visualized using iTOL (18). Phylum CSSED10-310 is highlighted in yellow, and all other phyla are wedged. The 5 MAGs from Zodletone Spring discussed here are shown in red bold text. Names depict the MAG bin name (as shown in Table 1). The single CSSED10-310 genome (assembly accession number GCA_003558985.1) available in GTDB is also highlighted in the same clade. The tree was midpoint rooted, and the bootstrap values (from 100) are displayed for the branches with ≥50% support.

Five genomes recovered from the spring source sediment metagenome were affiliated with the rare, yet-uncultured phylum CSSED10-310 (Fig. 1). Sequencing statistics (including the number of contigs, median genome coverage, and *N*_50_ value) and general genomic features of the CSSED10-310 genomes are shown in Table 1. The expected genome sizes ranged from 3.01 to 5.72 Mbp, and the GC content ranged from 43.4 to 58.9%. The cells are predicted to be Gram negative and possibly motile (based on the identification of the majority of flagellum and type IV pilus biosynthesis and assembly genes). A heterotrophic lifestyle is predicted, with sugars (glucose, fructose, mannose, ribulose, and galactose), starch, and propionate as potential carbon sources. Two genomes (Zod_Metabat.252 and Zod_Metabat.419) encoded the anaerobic sulfite reductase (AsrABC) system, as well as the membrane-bound heterodisulfide reductase-related enzymes (HdrABC) for transfer of electrons to the AsrC subunit, suggesting sulfite reduction capacities coupled to sugar degradation as an energy-generating process in the analyzed phylum CSSED10-310 genomes. In addition, the genomes encoded sugar fermentative capabilities.

**TABLE 1:** General genomic features of the five MAGs studied

	Data for indicated MAG bin name				
Parameter	Zod_Metabat.1153	Zod_Metabat.1246	Zod_Metabat.252	Zod_Metabat.419	Zod_Metabat.479
GenBank assembly accession no.	JAFGDC000000000	JAFGEQ000000000	JAFGJC000000000	JAFGLW000000000	JAFGMN000000000
Completeness (%)	86.32	85.42	68.11	82.85	85.59
Contamination (%)	4.75	0.85	0	1.28	5.13
GTDB classification					
Phylum	CSSED10-310	CSSED10-310	CSSED10-310	CSSED10-310	CSSED10-310
Class	CSSED10-310	CSSED10-310	CSSED10-310	CSSED10-310	CSSED10-310
Order	Novel order ZNO13^*a*^	CSSED10-310	CSSED10-310	CSSED10-310	CSSED10-310
Family	Novel family ZNF029^*b*^	CSSED10-310	Novel family ZNF028^*b*^	Novel family ZNF028^*b*^	CSSED10-310
No. of contigs	635	24	522	31	360
Assembly *N*_50_ (bp)	9,603	245,466	9,322	180,367	13,125
Median genome coverage (×)	9.54	30.39	8.98	25.83	9.59
Assembly size (Mbp)	4.94	2.57	3.85	3.12	3.46
Expected genome size (Mbp)	5.72	3.01	5.65	3.76	4.04
GC content (%)	58.9	48.9	43.4	54	53.2
No. of genes	4,044	2,073	3,368	2,515	2,993
Coding %	89.05	94.16	93.29	93.96	94.09
Avg gene length (bp)	1,087	1,168	1,066	1,165	1,087
No. of rRNA genes					
5S rRNA	1	1	0	1	1
16S rRNA	0	0	0	0	0
23S rRNA	0	0	0	0	0
No. of tRNA genes	34	35	18	41	30
Quality tiers	MQD^*c*^	MQD	MQD	MQD	MQD
CRISPR count	9	3	1	6	1

a Genomes were unclassified by the GTDB-Tk at the order level and were assigned to a novel order.

b Genomes were unclassified by the GTDB-Tk at the family level and were assigned to a novel family.

c MQD, medium quality draft.

### Data availability.

Raw sequencing reads were deposited in the SRA under accession number SRX9813571. The whole-genome shotgun project was submitted to GenBank under BioProject number PRJNA690107 and BioSample number SAMN17269717. The individual metagenome-assembled genomes (MAGs) have been deposited at DDBJ/ENA/GenBank under the accession numbers JAFGEQ000000000, JAFGDC000000000, JAFGJC000000000, JAFGMN000000000, and JAFGLW000000000 and were annotated using the NCBI Prokaryotic Genome Annotation Pipeline.
